# Comprehensive profiles and diagnostic value of menopausal-specific gut microbiota in premenopausal breast cancer

**DOI:** 10.1038/s12276-021-00686-9

**Published:** 2021-10-27

**Authors:** Ming-Feng Hou, Fu Ou-Yang, Chung-Liang Li, Fang-Ming Chen, Chieh-Han Chuang, Jung-Yu Kan, Cheng-Che Wu, Shen-Liang Shih, Jun-Ping Shiau, Li-Chun Kao, Chieh-Ni Kao, Yi-Chen Lee, Sin-Hua Moi, Yao-Tsung Yeh, Chien-Ju Cheng, Chih-Po Chiang

**Affiliations:** 1 Department of Surgery, Kaohsiung Medical University Hospital, Kaohsiung Medical University, Kaohsiung, 80756, Taiwan; 2 Division of Breast Oncology and Surgery, Department of Surgery, Kaohsiung Medical University Hospital, Kaohsiung Medical University, Kaohsiung, 80756 Taiwan; 3 Department of Biomedical Science and Environmental Biology, College of Life Science, Kaohsiung Medical University, Kaohsiung, 80756 Taiwan; 4 Graduate Institute of Medicine, College of Medicine, Kaohsiung Medical University, Kaohsiung, 80756 Taiwan; 5 Graduate Institute of Clinical Medicine, College of Medicine, Kaohsiung Medical University, Kaohsiung, 80756 Taiwan; 6 Department of Anatomy, School of Medicine, College of Medicine, Kaohsiung Medical University, Kaohsiung, 80756 Taiwan; 7 Center of Cancer Program Development, E-Da Cancer Hospital, I-Shou University, Kaohsiung, 82445 Taiwan; 8 Aging and Disease Prevention Research Center, Fooyin University, Kaohsiung, 83102 Taiwan; 9 Department of Medical Laboratory Sciences and Biotechnology, Fooyin University, Kaohsiung, 83102 Taiwan

**Keywords:** Bacterial genetics, Breast cancer

## Abstract

In Western countries, breast cancer tends to occur in older postmenopausal women. However, in Asian countries, the proportion of younger premenopausal breast cancer patients is increasing. Increasing evidence suggests that the gut microbiota plays a critical role in breast cancer. However, studies on the gut microbiota in the context of breast cancer have mainly focused on postmenopausal breast cancer. Little is known about the gut microbiota in the context of premenopausal breast cancer. This study aimed to comprehensively explore the gut microbial profiles, diagnostic value, and functional pathways in premenopausal breast cancer patients. Here, we analyzed 267 breast cancer patients with different menopausal statuses and age-matched female controls. The α-diversity was significantly reduced in premenopausal breast cancer patients, and the β-diversity differed significantly between breast cancer patients and controls. By performing multiple analyses and classification, 14 microbial markers were identified in the different menopausal statuses of breast cancer. *Bacteroides fragilis* was specifically found in young women of premenopausal statuses and *Klebsiella pneumoniae* in older women of postmenopausal statuses. In addition, menopausal-specific microbial markers could exhibit excellent discriminatory ability in distinguishing breast cancer patients from controls. Finally, the functional pathways differed between breast cancer patients and controls. Our findings provide the first evidence that the gut microbiota in premenopausal breast cancer patients differs from that in postmenopausal breast cancer patients and shed light on menopausal-specific microbial markers for diagnosis and investigation, ultimately providing a noninvasive approach for breast cancer detection and a novel strategy for preventing premenopausal breast cancer.

## Breast cancer: gut microbes before and after menopause

Women developing breast cancer before the menopause have less diverse gut microbe populations than their healthy peers, and significantly different gut microbial profiles than postmenopausal breast cancer patients. There is increasing evidence of a link between gut microbes and breast cancer, but most studies have focused on women after the menopause. The incidence of breast cancer in premenopausal women is increasing, especially in Asia. Researchers in Taiwan led by Chih-Po Chiang at Kaohsiung Medical University Hospital compared gut microbial populations in 267 breast cancer patients at different menopausal stages to matched controls. The results suggest that gut microbe profiling might offer a novel and noninvasive method for diagnosing and monitoring breast cancer. It may also lead to new strategies for prevention and treatment, as evidence emerges of a link between gut microbes and cancer.

## Introduction

Breast cancer is the most commonly diagnosed cancer worldwide, with an estimated 2.3 million incident cases (11.7%) reported in 2020. Breast cancer is also the fifth leading cause of cancer mortality worldwide and the leading cause of cancer-associated death in women^1^. Compared to the incidence of breast cancer in Western countries, the incidence of breast cancer in Asia is relatively low; however, the proportion of younger women diagnosed with breast cancer is increasing in Asian countries. The median onset age of breast cancer in Asia (40–50 years) is approximately 20 years younger than that in Western countries (60–70 years)^2,3^. In Taiwan, more than 30% of women with breast cancer are younger than 50 years of premenopausal breast cancer. This high proportion of premenopausal breast cancer in Taiwan differs from that in Western countries, where the proportion is usually less than 20%^4,5^.

Genetic background is one of the potential explanations of this premenopausal status of young breast cancer patients. Germline mutations in *BRCA1* and *BRCA2* are the dominant targets in this subtype of young breast cancer patients. Based on multiple gene analyses, *TP53* and *RAD50* are also involved in the pathogenesis of breast cancer in young patients^6^. However, *BRCA* and *TP53* mutations are present in only 20% of young breast cancer patients, especially in those with the familial type. Westernization might play an important role in Asia of young breast cancer patients, including environmental (e.g., endocrine-disrupting chemicals, EDCs), dietary (e.g., high fat and alcohol intake), and reproductive factors (e.g., delayed childbearing)^7,8^. However, the role of the gut microbiota in premenopausal breast cancer has often been neglected.

The gut microbiota is a collection of information on the bacteria in our gut, including the number, type, diversity, etc. The gastrointestinal tract contains >10^14^ microorganisms, which is ten times the number of our own cells, and accounts for 1–2 kg of body weight. The gut microbiota has been well recognized as a symbiotic organ that maintains the normal function of the gut and affects metabolic, protective, and structural functions^9^. Dysbiosis, a condition that reflects the imbalance of the gut microbiota, has been recognized to be closely associated with many human diseases, including colorectal cancer^10^, metabolic syndrome^11^, inflammatory bowel disease^12^, and brain nerve diseases^13^. The gut microbiota is also involved in cancer formation, and is termed the “oncobiome”, which induces the transformation of host cells and accounts for 20% of human malignancies^14^.

To date, the best-known mechanism by which the gut microbiota affects breast cancer is through the “estrobolome”, which refers to gut bacterial genes (gut microbiota) whose products can metabolize estrogens^15,16^. The metabolism of conjugated estrogens occurs in the liver, and the compounds are excreted in urine, bile, and feces; however, conjugated estrogens can be deconjugated by gut bacteria via β-glucuronidase activity, and estrogen can be reabsorbed into circulation. The accumulation of endogenous estrogens increases the risk of breast cancer, especially in postmenopausal women^17^. In addition to the estrobolome, bacterial metabolites are transferred to distant sites through circulation and influence the occurrence of breast cancer^18^. Thus, the gut microbiota secretes and synthesizes bioactive metabolites that affect breast cancer formation.

However, the concept of the estrobolome mainly exists in postmenopausal breast cancer, and bacterial metabolites were verified in a cell line model. In addition, several human studies have focused on the gender or menopausal status of healthy controls, but not of breast cancer patients. Moreover, only a few studies have assessed the gut microbiota in premenopausal breast cancer patients. Thus, in this study, we aimed to comprehensively explore the gut microbial profiles between premenopausal and postmenopausal breast cancer patients to elucidate the critical microbial markers, diagnostic value, and related functional pathways in premenopausal breast cancer patients, ultimately providing a noninvasive approach for breast cancer detection and a novel strategy for preventing premenopausal breast cancer in the future.

## Materials and methods

### Patient recruitment

From October 2018 to December 2020, 267 subjects were recruited from the Division of Breast Oncology and Surgery, Department of Surgery, Kaohsiung Medical University Chung-Ho Memorial Hospital. The subjects included 67 age-matched female controls (premenopausal, Pre-C = 50; postmenopausal, Post-C = 17) and 200 breast cancer patients (premenopausal, Pre-BC = 100; postmenopausal, Post-BC = 100). All patients with *de novo* breast cancer were diagnosed with stage I–II disease by pathological examination. Subjects were excluded if they were diagnosed with malignancies other than breast cancer or administered steroids, antibiotics, or probiotics within 4 weeks before the screening visit. Fecal samples were collected for DNA extraction before patients received any chemo/hormone/target therapy, radiation, or surgery. Furthermore, clinical characteristics such as age, body mass index, grade, stage, tumor size, and receptor status were recorded (Table 1). The study was approved by the Internal Review Board, and informed consent was obtained from all subjects [KMUHIRB-G(II)-20180018 and KMUHIRB-E(I)-20200285].

**Table 1:** Clinical characteristics of subjects according to the menopausal status.

Characteristic	Pre-C *N* = 50	Pre-BC *N* = 100	Post-C *N* = 17	Post-BC *N* = 100	*p*
Age (m ± sd)	35.4 ± 6	41.5 ± 5.2	61.6 ± 8.9	60.08 ± 5.8	<0.001
BMI		23.6 ± 4.4		24.3 ± 4.2	0.209
Grade					0.261
Grade 1	–	10 (10.0%)	–	7 (7.0%)	
Grade 2	–	60 (60.0%)	–	71 (71.0%)	
Grade 3	–	30 (30.0%)	–	22 (22.0%)	
Stage					0.657
Stage I	–	63 (63.0%)	–	66 (66.0%)	
Stage II	–	37 (37.0%)	–	34 (34.0%)	
Tumor size (mm)	–	24.9 ± 15.4	–	21.3 ± 10.2	0.049
ER					0.182
–	–	13 (13.0%)	–	20 (20.0%)	
+	–	87 (87.0%)	–	80 (80.0%)	
PR					0.008
–	–	20 (20.0%)	–	37 (37.0%)	
+	–	80 (80.0%)	–	63 (63.0%)	
HER2					0.102
–	–	80 (80.0%)	–	70 (70.0%)	
+	–	20 (20.0%)	–	30 (30.0%)	
Ki67					0.199
–	–	39 (39.0%)	–	48 (48.0%)	
+	–	61 (61.0%)	–	52 (52.0%)	

*p* value: Comparison between Pre-BC and Post-BC with *t*-test and Chi-squared test.

### Fecal DNA Extraction and 16S sequencing

DNA was extracted according to the manufacturer’s instructions using a QIAamp Fast DNA Stool Mini Kit (Qiagen, Valencia, CA, USA) to obtain an OD 260/280 ratio between 1.8 and 2.0 by NanoDrop 2000 spectrophotometer. The V3-V4 hypervariable region of 16S rDNA was amplified using the bacterial-specific forward (5′ TCGTCGGCAGCGTCAGATGTGTATAAGAGACAGCCTACGGGNGGCWGCAG 3′) and reverse (5′ GTCTCGTGGGCTCGGAGATG TGTATAAGAGACAGGACTACHVGGGTATCTAATCC 3′) primer sets. The PCR product was purified with AMPure XP magnetic beads (Beckman Coulter, Brea, IN, USA) and indexed adapters were added to the amplicons using the Nextera XT Index Kit (Illumina, San Diego, CA, USA) according to the manufacturer’s instructions. The amplified DNA sizing accuracy was verified using the 4200 TapeStation System (Agilent Technologies, Santa Clara, CA, USA). After library construction, samples were mixed with MiSeq Reagent Kit v3 (600-cycle) and loaded onto a MiSeq cartridge. Then, a 2 × 300 bp paired-end sequencing run was performed using the MiSeq platform (Illumina, San Diego, CA, USA).

### Taxonomic composition and diversity analysis

The raw paired-end reads were trimmed and passed through quality filters (quality trimming, discarding short read length, and removing chimeras) and were assigned to operational taxonomic units (OTUs) that shared ≥97% similarity with the Greengene 13.8 database. Raw paired-end reads were also analyzed using the BaseSpace Ribosomal Database Project (RDP) classifier. OTUs (relative abundance), α-diversity (Shannon entropy and Venn diagram), and β-diversity (PCoA-D_0.5 UniFrac) were determined using BaseSpace (Illumina, San Diego, CA, USA), CLC Genomics Workbench 21 with Microbial Genomics Module (Qiagen, Germantown, MD, USA) and GraphPad Prism 8 (GraphPad Software, La Jolla, CA, USA)^19^.

### Linear discriminant analysis (LDA) effect size (LEfSe) algorithm

The LDA effect size (LEfSe) algorithm with α = 0.05 (Kruskal–Wallis and Wilcoxon tests) and effect size threshold of 2 on LDA was used to identify taxa of contrasting abundance between groups. The LEfSe identified taxa that (1) were of nonzero abundance in more than half of the samples in at least one comparison group, and (2) had an abundance difference > 0.0001 between the maximum and minimum group means were labeled on the LEfSe cladogram. Significant genera and species were further investigated for LDA scores and relative abundance by bar plots and box plots, respectively, if available.

### Heatmap analysis

To visualize how bacterial abundance and samples were clustered, heatmaps were constructed using the R package ‘pheatmap’ at the genus and species levels, including genera with an average relative abundance > 0.5% and species > 0.05%. Taxa with “uncultured” or “unidentified” names were excluded. Hierarchical clustering in the heatmap was performed using rows and columns based on Euclidean distances with the complete linkage algorithm.

### Correlation matrix analysis

Pairwise correlation analysis was performed among taxonomic groups at the genus level. Genera with an average relative abundance > 0.5% were included, and those with “uncultured” or “unidentified” taxa were excluded. The correlation was evaluated using Spearman’s rank correlation and the cutoff was set at 0.2. The correlation matrix was visualized using the R package ‘ggcorrplot’, in which positive correlations were colored blue and negative correlations were colored red.

### Functional profile inference and analysis

The metabolic functional profile of the microbiota was predicted using the PICRUSt2 algorithm based on OTU-level read abundance. The predicted abundance of functional groups, including the KEGG Orthology (KO) and MetaCyc pathways was normalized by total read counts per sample for downstream analysis. The KO pathway (KOPath) abundance was also calculated as the sum of the involved KO abundances and normalized as mentioned for downstream analysis. Differential enrichment analysis was performed using the Kruskal–Wallis test. The results were first visualized by a volcano plot showing false discovery rate (FDR, in log 10) versus fold changes (in log 2) between groups. The functional pathways with significant differences were further explored for abundance distribution using a boxplot for those with >50% of samples with nonzero abundance in at least one compared group, and reported in descending order of group mean differences; that is, the greater the difference, the higher the priority.

### Statistical analysis

Comparisons of different groups were performed using the two-tailed *t*-test and one-way ANOVA. Values of *p* less than *p* < 0.05, *p* < 0.01, and *p* < 0.001 were considered statistically significant. The specificity and sensitivity of the microbial markers were determined using the receiver operating characteristic curve (ROC curve) and the area under the curve (AUC) values. Correlations were calculated using the Pearson correlation coefficient. All statistical analyses were performed using GraphPad Prism 8 (GraphPad Software, La Jolla, CA, USA)^19^.

## Results

### Difference in diversity and taxonomy of gut microbiota between control individuals and breast cancer patients with different menopausal statuses

In this study, we recruited 267 participants belonging to four groups: premenopausal female controls (Pre-C, *N* = 50), premenopausal breast cancer patients (Pre-BC, *N* = 100), postmenopausal female controls (Post-C, *N* = 17), and postmenopausal breast cancer patients (Post-BC, *N* = 100). The clinical characteristics of the individuals in the four groups are summarized in Table 1. Patients in the Pre-BC and Post-BC groups had similar body mass indices (BMIs) and grades; however, a significant difference was found in tumor size and progesterone receptor (PR) status.

**Fig. 1:**
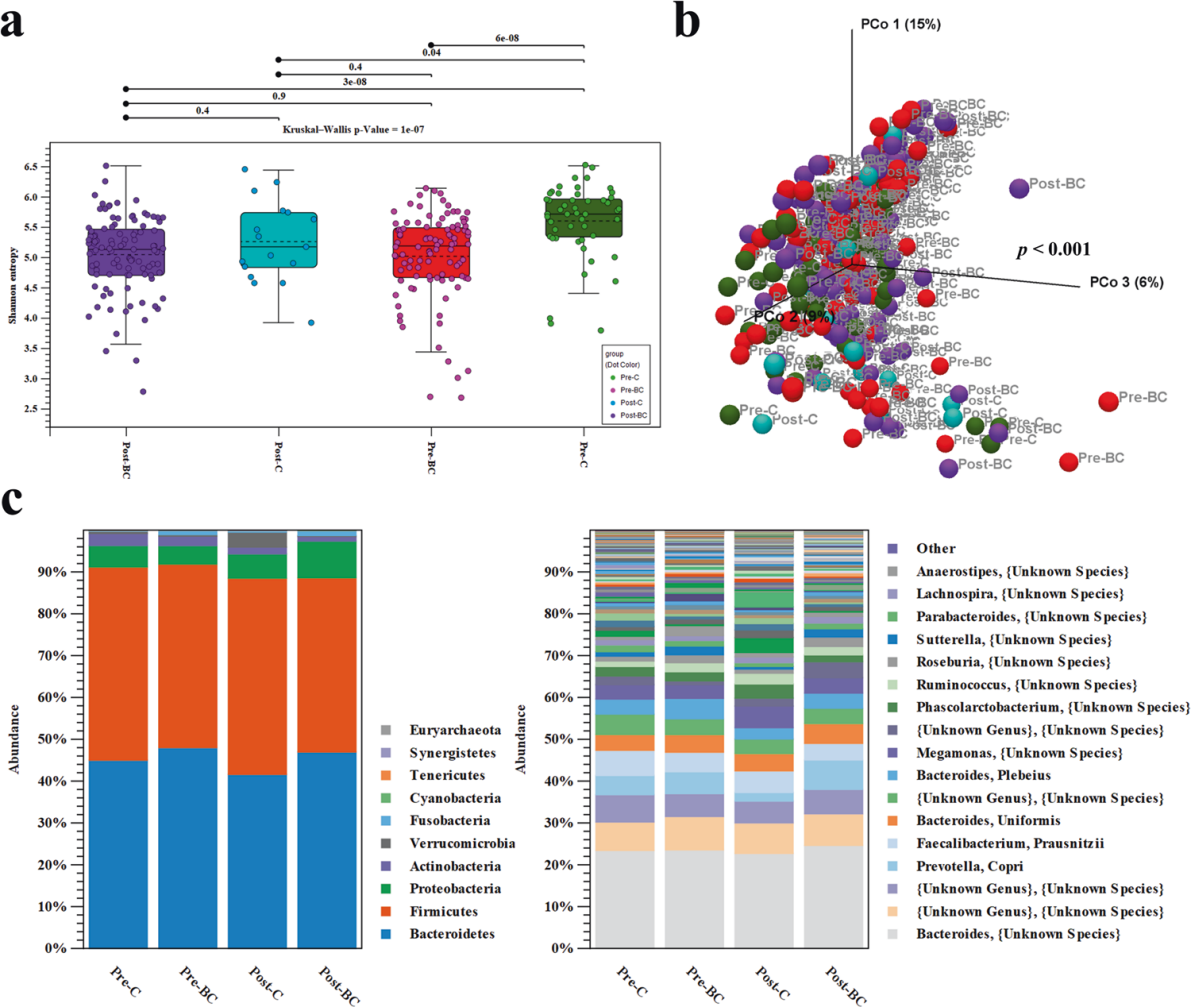
Microbial diversity among different groups of control individuals and breast cancer patients. **a** The α-diversity of Shannon entropy in Pre-BC (red box) was significantly lower than that in Pre-C (green box); this phenomenon was not observed in Post-BC (purple box). **b** The β-diversity of PCoA demonstrated significant differences (*p* < 0.001) in the total microbial composition among the four groups. **c** The relative abundance of OTUs at the phylum and species levels among the four groups.

Initially, we analyzed the α- and β-diversities among the four groups. The α-diversity of Shannon entropy was significantly reduced in the Pre-BC group (red box) compared to in the Pre-C group (green box); however, no significant differences were found in α-diversity between the Post-C (blue box) and Post-BC groups (purple box) or the Pre-BC and Post-BC groups (Fig. 1a). In terms of β-diversity, principal coordinate analysis (PCoA) of D_0.5 UniFrac was performed to determine the total microbial composition of the different groups. The PERMANOVA test revealed a significant difference in the overall microbial composition among the different groups (*p* < 0.001, Fig. 1b). To further explore the microbial composition, the relative abundances of operational taxonomic units (OTUs) were evaluated among the different groups. At the phylum and species levels, *Actinobacteria* was enriched in Pre-C, whereas *Verrucomicrobia* was enriched in Post-C and *Proteobacteria* in Post-BC (Fig. 1c). These results indicate that α-diversity was specifically decreased in premenopausal breast cancer patients. In addition, the overall microbial composition was significantly different between control individuals and breast cancer patients.

### Identification of the microbial markers of the different groups

To further reveal the microbial markers of the different groups, LDA effect size (LEfSe), Venn diagram, and heatmap analyses were used to identify (1) critical microbial markers of premenopausal breast cancer without age effects; (2) microbial markers that fluctuated with age but were more obviously altered in breast cancer; and (3) universal microbial markers of breast cancer.

**Fig. 2:**
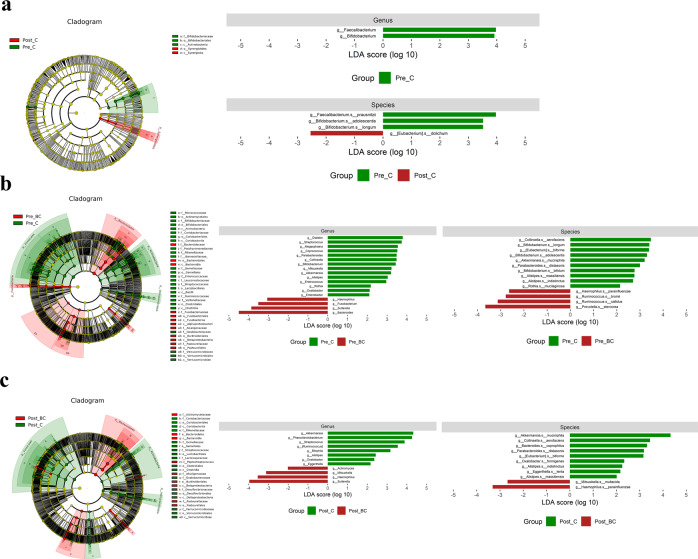
Microbial markers at the genus/species levels between control individuals and breast cancer patients. **a** The potential microbial markers between Pre-C and Post-C. **b** The potential microbial markers between Pre-C and Pre-BC. **c** The potential microbial markers between Post-C and Post-BC.

**Fig. 3:**
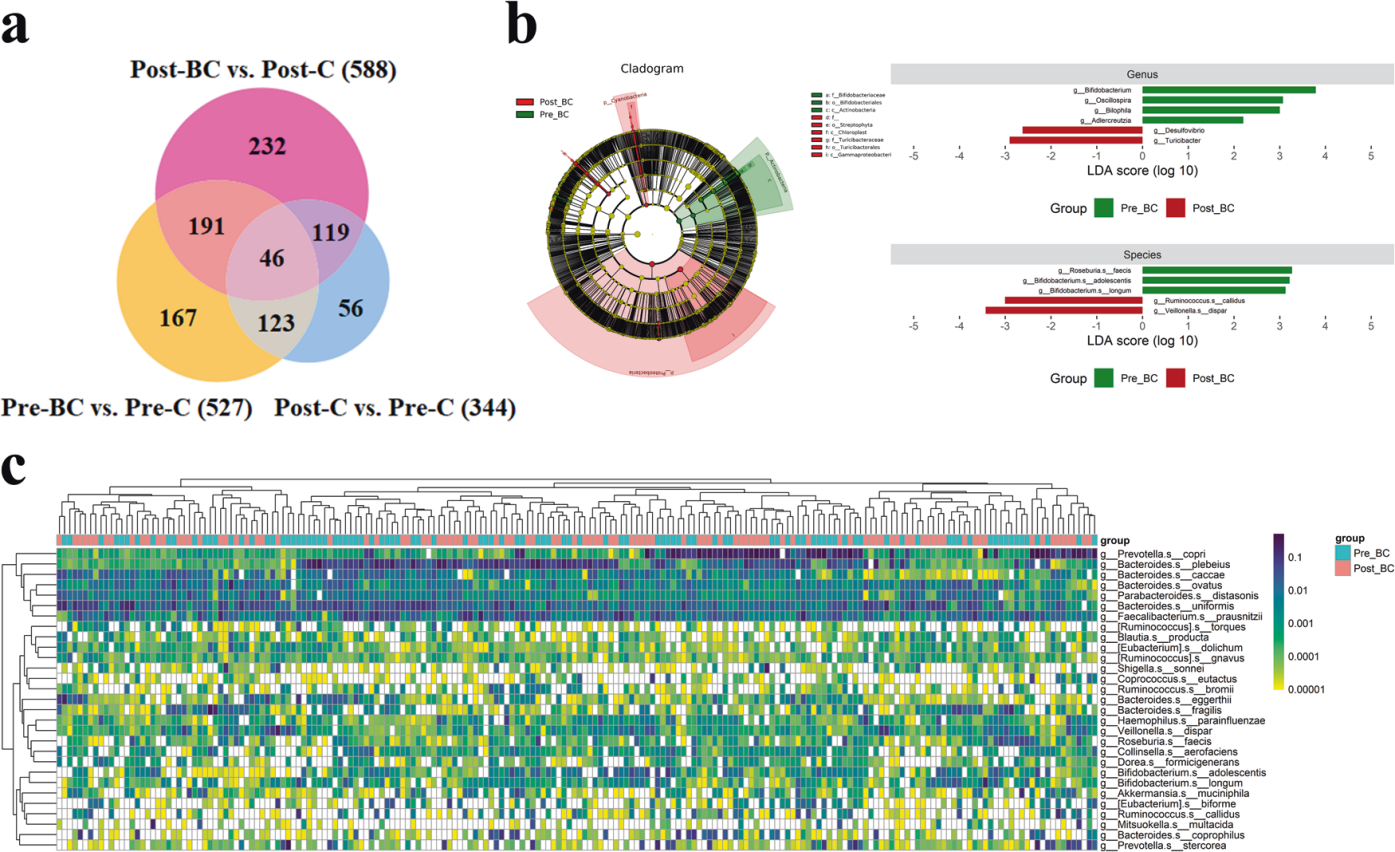
The intersection of OTUs among different groups of control individuals and breast cancer patients. **a** The Venn diagram analysis shows that 167 OTUs belonged to Pre-BC versus Pre-C and 191 OTUs belonged to breast cancer (overlap of Pre-BC and Post-BC). **b**, **c** The potential microbial markers between Pre-BC and Post-BC.

We specifically focused on the genus/species levels among the different groups. Based on LEfSe analysis, *Faecalibacterium* and *Bifidobacterium* were identified to be more enriched in Pre-C than in Post-C. When Pre-C was compared with Pre-BC and Post-C with Post-BC, *Bifidobacterium* and *Akkermansia* were enriched in control individuals, while *Sutterella* and *Haemophilus* were enriched in breast cancer patients (Fig. 2). A Venn diagram was subsequently generated to verify the intersection of OTUs among the different groups with the criteria of absolute fold change ≧ 5 and *p* < 0.05 (Fig. 3a). We found 167 OTUs belonging to Pre-BC versus Pre-C and 191 OTUs belonging to breast cancer (overlap of Pre-BC and Post-BC). We also compared Pre-BC with Post-BC with LEfSe and heatmap analyses to identify differential microbiomes (Fig. 3b, c). Accordingly, several potential microbial markers among the different groups based on LEfSe, Venn diagram, and heatmap analyses were selected as candidates for further analysis.

### Unique gut microbial markers of different menopausal statuses of breast cancer

To further verify the gut microbial markers of different menopausal statuses, we utilized BaseSpace in the Ribosomal Database Project (RDP) classifier to reconfirm the OTUs at the genus/species levels. Using the RDP classifier, we screened all of the potential microbial markers of OTUs obtained via LEfSe, Venn diagram, and heatmap analyses at the genus/species levels and eliminated the microbiomes with low percentages or that were not present in statistically significant numbers in the four groups of Pre-C, Pre-BC, Post-C, and Post-BC. Eventually, we identified 14 bacterial taxa that were abundant and/or present in statistically significant quantities in the four groups.

**Fig. 4:**
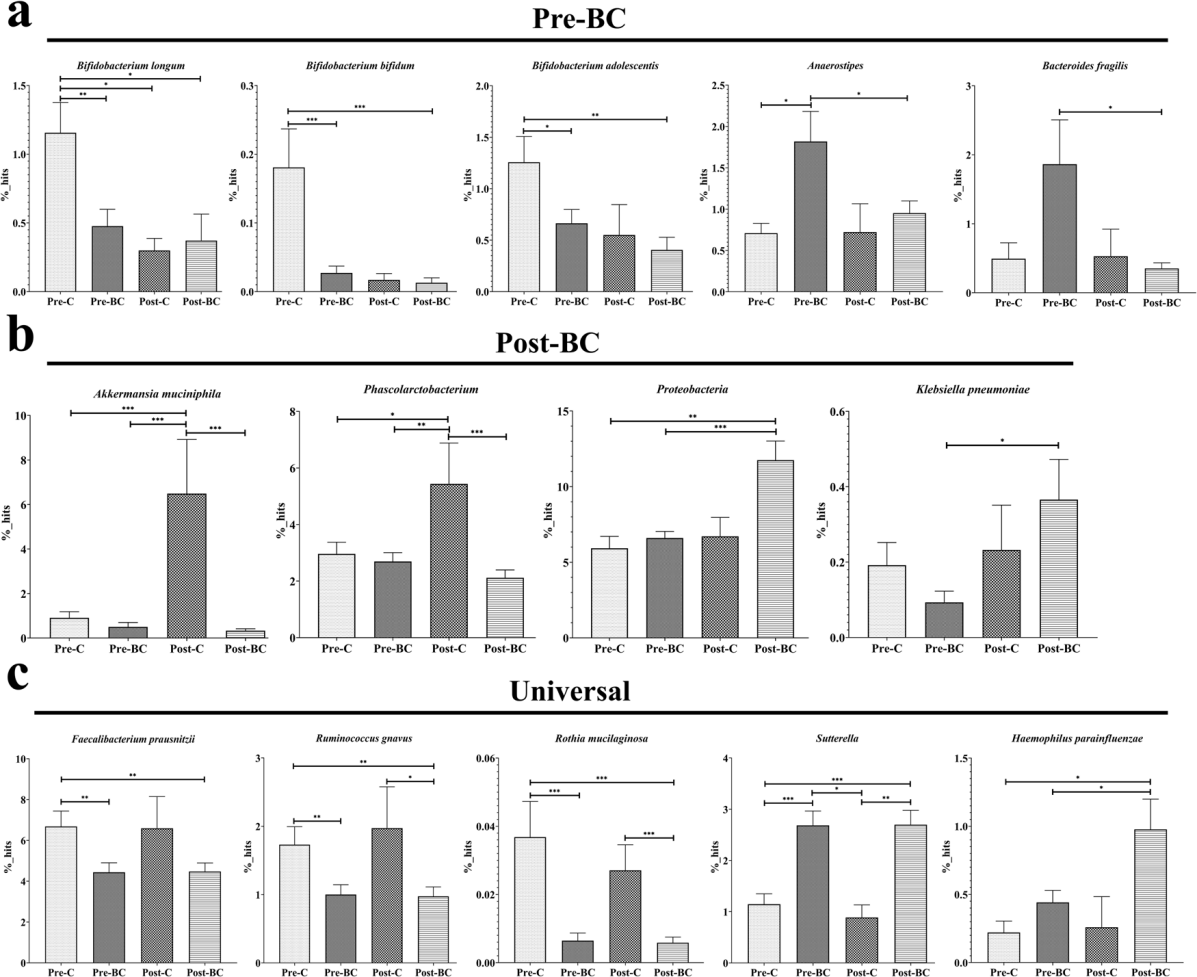
The 14 microbial markers according to the different menopausal statuses of breast cancer. **a** The percentage of *Bifidobacterium* spp. fluctuated with age but was significantly reduced in Pre-BC compared with Pre-C, whereas *Anaerostipes* and *Bacteroides fragilis* were specifically increased in Pre-BC. **b** The percentages of *Akkermansia muciniphila* and *Phascolarctobacterium* fluctuated with age but were significantly reduced in Post-BC compared with Post-C, whereas *Proteobacteria* and *Klebsiella pneumoniae* were specifically increased in Post-BC. **c** The percentages of *Faecalibacterium prausnitzii, Ruminococcus gnavus*, and *Rothia mucilaginosa* were simultaneously reduced in Pre-BC, and Post-BC, while *Sutterella*, and *Haemophilus parainfluenzae* were simultaneously increased in Pre-BC and Post-BC.

The 14 bacterial taxa were divided into three groups: Pre-BC, Post-BC, and Universal. The abundances of *Bifidobacterium longum, Bifidobacterium bifidum*, and *Bifidobacterium adolescentis* were found to fluctuate with age but were more obviously reduced in premenopausal breast cancer patients. The abundance of *Bifidobacterium* spp. was higher in Pre-C but lower in Post-C due to the age effect; however, *Bifidobacterium* spp. was significantly reduced in Pre-BC compared with Pre-C individuals; this phenomenon was not observed in Post-BC patients. We also found that *Anaerostipes* and *Bacteroides fragilis* were not affected by age and were significantly higher in premenopausal breast cancer patients than in postmenopausal breast cancer patients (Fig. 4a). Thus, the above five bacterial taxa were unique to premenopausal breast cancer patients, with or without the age effect. In Post-BC, we found that the abundances of *Akkermansia muciniphila* and *Phascolarctobacterium* fluctuated with age but were more obviously reduced in postmenopausal breast cancer patients. *Akkermansia muciniphila* and *Phascolarctobacterium* were higher in Post-C individuals than in Pre-C individuals due to the age effect; however, they were significantly reduced in Post-BC patients compared with their levels in Post-C individuals; this phenomenon was not observed in Pre-BC patients. We also found that *Proteobacteria* and *Klebsiella pneumoniae* were not affected by age, with significantly higher levels in postmenopausal breast cancer patients than in premenopausal breast cancer patients (Fig. 4b). Thus, the above four bacterial taxa were unique to postmenopausal breast cancer patients with or without the age effect.

In the Universal group, the abundances of *Faecalibacterium prausnitzii, Ruminococcus gnavus*, and *Rothia mucilaginosa* were simultaneously reduced in Pre-BC and Post-BC patients, while *Sutterella* and *Haemophilus parainfluenzae* were simultaneously increased in Pre-BC and Post-BC patients (Fig. 4c). Thus, the above five bacterial taxa were universal microbiomes for breast cancer patients (for both premenopausal and postmenopausal breast cancer) without age effects. The above results indicate that the gut microbiome is unique according to different menopausal statuses of breast cancer. We also verified the above bacterial taxa at the genus/species level in individuals with different receptor statuses and stages. The 13 bacterial taxa did not show a significant trend among the different receptor statuses (luminal, HER2, basal) or stages (stages 1, 2), with only a small proportion of bacterial taxa reaching statistical significance (Supplementary Figs. 1, 2).

### Certain microbial markers correlate with age in control individuals and breast cancer patients

**Fig. 5:**
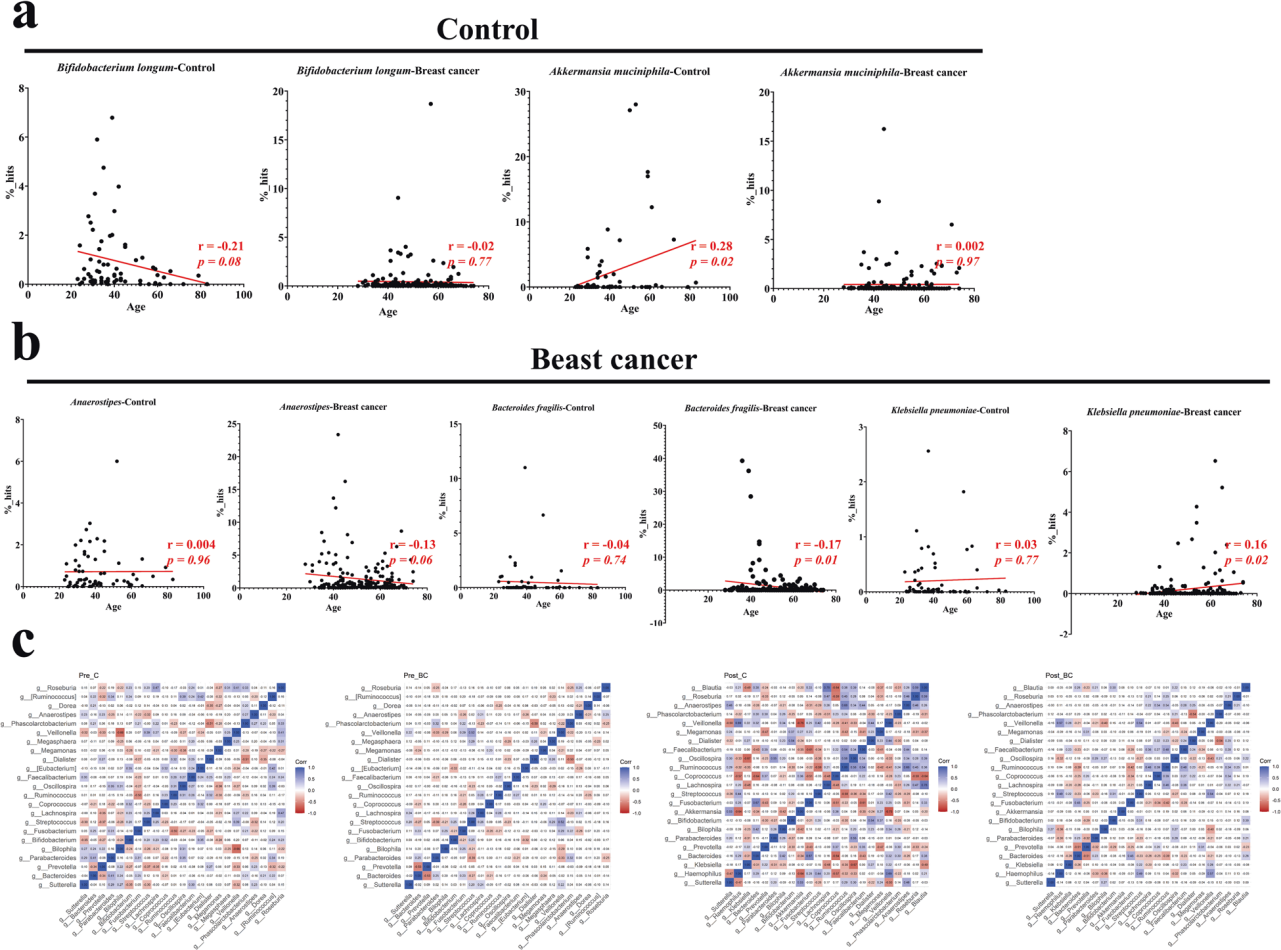
Critical microbial markers that correlate with age in control individuals and breast cancer patients. **a** *Bifidobacterium longum* was negatively correlated (*r* = −0.21, *p* = 0.08) and *Akkermansia muciniphila* was positively correlated (*r* = 0.28, *p* = 0.02) with age in female controls but did not correlate with age in breast cancer patients. **b** *Anaerostipes* (*r* = −0.13, *p* = 0.06) and *Bacteroides fragilis* (*r* = −0.17, *p* = 0.01) were negatively correlated with age, while *Klebsiella pneumoniae* was positively correlated (*r* = 0.16, *p* = 0.02) with age in breast cancer patients but did not correlate with age in female controls. **c** Spearman correlation of Pre-C, Pre-BC, Post-C, and Post-BC at the genus level.

To further determine the microbial markers that are crucial in breast cancer with age fluctuation, especially in premenopausal of young breast cancer. We analyzed the correlation between 14 bacterial taxa and age in two separate groups: control (Pre-C + Post-C) and breast cancer (Pre-BC + Post-BC). Among the 14 bacterial taxa, *Bifidobacterium longum* was negatively correlated (*r* = −0.21, *p* = 0.08) and *Akkermansia muciniphila* was positively (*r* = 0.28, *p* = 0.02) correlated with age in the control group but not in the breast cancer group (Fig. 5a), indicating that *Bifidobacterium longum* and *Akkermansia muciniphila* fluctuated with age in female controls but were not involved in the different menopausal statuses of breast cancer. In contrast, we found that *Anaerostipes* (*r* = −0.13, *p* = 0.06) and *Bacteroides fragilis* (*r* = −0.17, *p* = 0.01) were negatively correlated and *Klebsiella pneumoniae* was positively correlated (*r* = 0.16, *p* = 0.02) with age in the breast cancer group but not in the control group (Fig. 5b), indicating that *Anaerostipes, Bacteroides fragilis*, and *Klebsiella pneumoniae* were microbiomes that were specifically involved in the different menopausal statuses of breast cancer.

The correlations among the taxonomic groups was also verified by correlation matrix analysis to determine the effect of the dominant genus in female controls. In Pre-C, *Bifidobacterium* was negatively correlated with *Sutterella* (−0.35) and *Anaerostipes* (−0.11), thereby indicating the potential protective effect of *Bifidobacterium* in premenopausal female controls (Fig. 5c, left). On the other hand, in Post-C, *Akkermansia* and *Phascolarctobacterium* were negatively correlated with *Haemophilus* (*Akkermansia*: −0.64; *Phascolarctobacterium*: −0.22) and *Klebsiella* (*Akkermansia*: −0.12; *Phascolarctobacterium*: −0.17), indicating a potential protective effect of *Akkermansia* and *Phascolarctobacterium* in postmenopausal female controls (Fig. 5c, right).

### Diagnostic value of the gut microbiota in the different menopausal statuses of breast cancer

In addition to elucidating the specific microbial markers in the different menopausal statuses of breast cancer, we sought to determine the potential diagnostic value of the gut microbiota for breast cancer. As specific microbial markers existed in premenopausal and postmenopausal breast cancer, the diagnostic value was verified by the menopausal status of three groups: premenopausal, postmenopausal, and all breast cancer patients (premenopausal and postmenopausal).

**Fig. 6:**
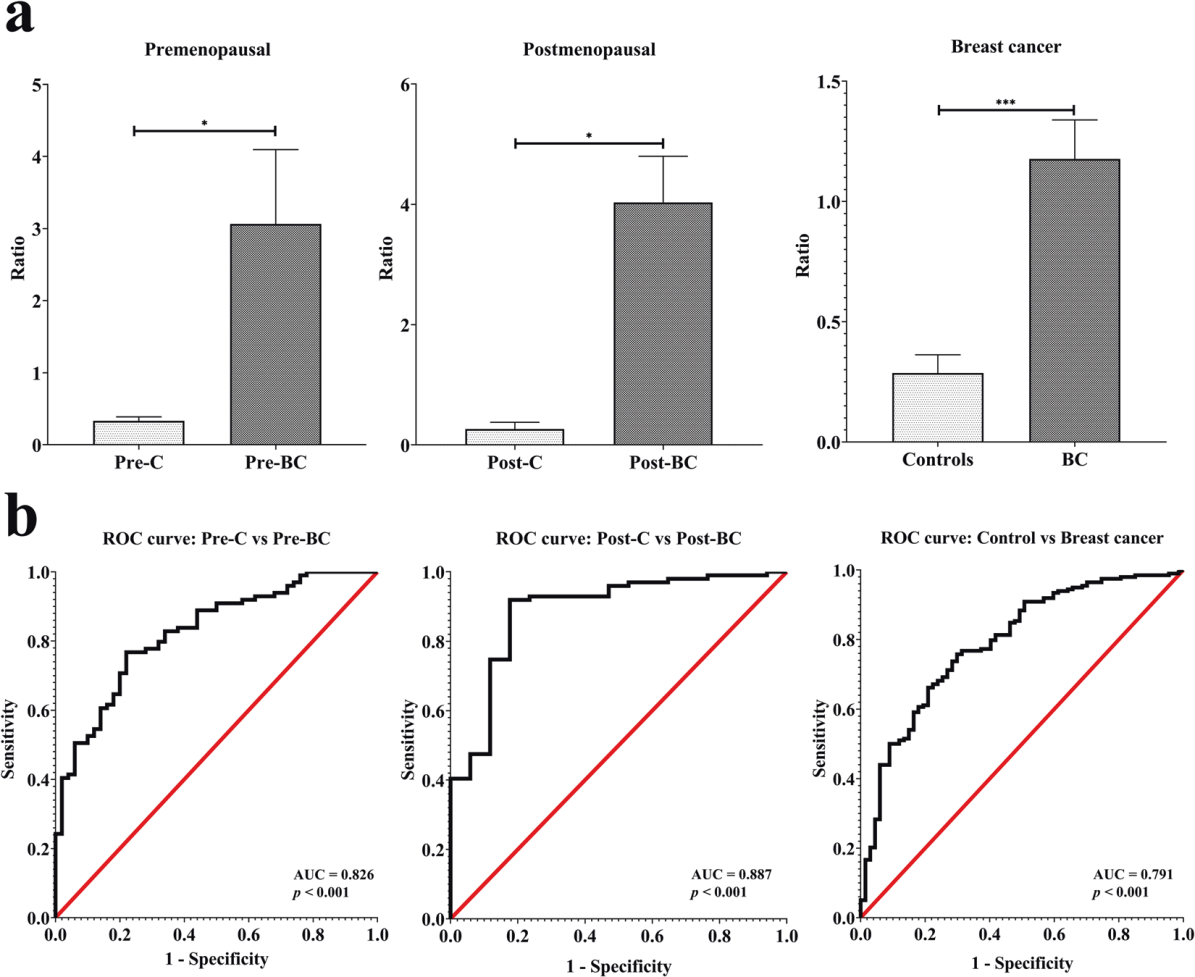
The potential of diagnosis using the microbial markers found in the different menopausal statuses of breast cancer. **a**, **b** Microbial markers were determined for the different menopausal statuses of breast cancer or all breast cancer (Pre-BC + Post-BC) by dividing the increasing taxa by the decreasing taxa. The average values displayed excellent discrimination for use in distinguishing Pre-BC/Post-BC from Pre-C/Post-C.

Regarding premenopausal status, ten bacterial taxa were calculated by dividing the increasing taxa by the decreasing taxa. The percentage of the sum (*Bacteroides fragilis* + *Anaerostipes* + *Haemophilus parainfluenzae* + *Sutterella*) divided by the sum (*Faecalibacterium prausnitzii* + *Bifidobacterium adolescentis* + *Bifidobacterium longum* + *Bifidobacterium bifidum* + *Ruminococcus gnavus* + *Rothia mucilaginosa*) yielded an average value (Pre-C: 0.33 versus Pre-BC: 3.06, *p* = 0.03). This average value was further evaluated using a receiver operating characteristic curve (ROC curve). The area under the curve (AUC) was 0.826 (*p* < 0.001), indicating excellent discrimination for distinguishing between Pre-BC and Pre-C (Fig. 6a, b left). For postmenopausal status, seven bacterial taxa were calculated by dividing the percentage of the sum (*Klebsiella pneumoniae* + *Haemophilus parainfluenzae* + *Sutterella*) by the sum (*Akkermansia muciniphila* + *Phascolarctobacterium* + *Ruminococcus gnavus* + *Rothia mucilaginosa*) to obtain an average value (Post-C: 0.27 versus Post-BC: 4.03, *p* = 0.02). For this average value, the AUC was 0.887 (*p* < 0.001), indicating excellent discrimination for distinguishing Post-BC from Post-C (Fig. 6a, b middle). In breast cancer patients regardless of menopausal status (premenopausal + postmenopausal), five bacterial taxa were calculated by dividing the percentage of the sum (*Haemophilus parainfluenzae* + *Sutterella*) by the sum (*Faecalibacterium prausnitzii* + *Ruminococcus gnavus* + *Rothia mucilaginosa*) to obtain an average value (Controls: 0.29 versus Breast cancer: 1.18, *p* < 0.001). For this average value, the AUC was 0.791 (*p* < 0.001), indicating acceptable discrimination for distinguishing breast cancer patients from control individuals (Fig. 6a, b right). The above results indicate that premenopausal and postmenopausal breast cancer differ in terms of the gut microbiota, which indicates diagnostic value for breast cancer.

### Metabolic functional pathway of the gut microbiota in breast cancer

**Fig. 7:**
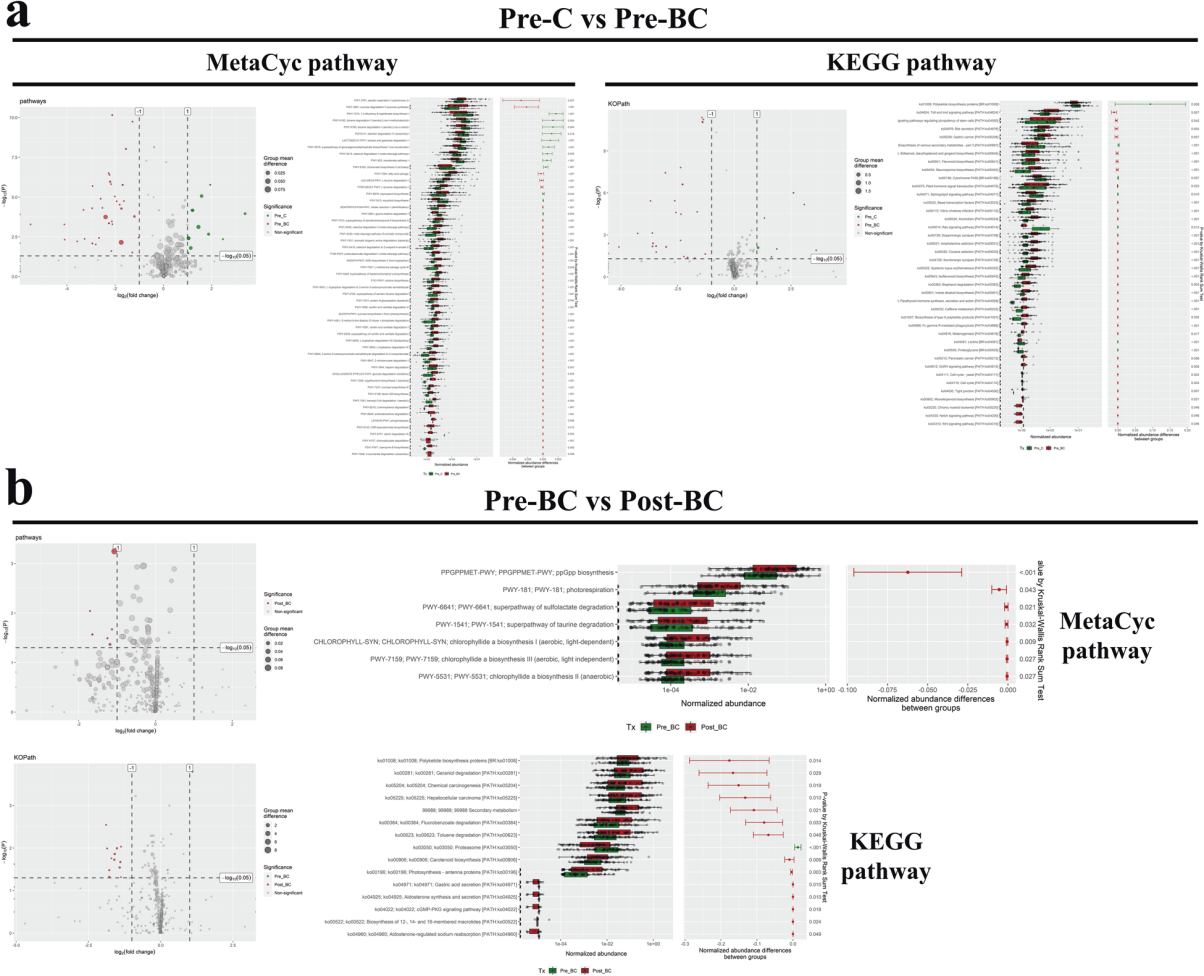
The potential functional pathway involved in breast cancer. **a** Compared with the control individuals, premenopausal breast cancer was enriched with pathways contributing to the abundance of the microbiome against the steroid-related (MetaCyc pathway) and oncogenic-related pathway (KEGG pathway). **b** Compared with premenopausal breast cancer patients, postmenopausal breast cancer patients exhibited greater enrichment in the steroid-related pathway (KEGG pathway).

To further understand the function of the microbiome in breast cancer, we employed PICRUSt2 prediction of the abundance of different microbiomes against MetaCyc (pathways) and KEGG pathways (KOPath). We specifically focused on premenopausal breast cancer to elucidate functional pathways of the gut microbiota. Compared with the control (Pre-C vs. Pre-BC), premenopausal breast cancer was enriched with pathways contributing to the abundance of the microbiome against the steroid-related (MetaCyc pathway: meta cleavage pathway of aromatic compounds; aromatic biogenic amine degradation; androstenedione degradation, Fig. 7a left) and oncogenic-related pathways (KEGG pathway: Cell cycle, Tight junction, Notch/Wnt signaling pathway, Fig. 7a right). We also compared premenopausal patients with postmenopausal breast cancer patients (Pre-BC vs. Post-BC) to verify the functional pathways of the microbiome in different menopausal statuses of breast cancer. Interestingly, we found that postmenopausal breast cancer patients exhibited greater enrichment of steroid-related (KEGG pathway: aldosterone synthesis and secretion; aldosterone−regulated sodium reabsorption) and chemical carcinogenesis pathways than premenopausal breast cancer patients (Fig. 7b).

## Discussion

Increasing evidence has suggested that the gut microbiota plays a critical role in cancer, which is termed the “oncobiome” and can drive cancer initiation and progression. The mechanisms of the oncobiome that contribute to carcinogenesis are divided into three categories: (1) indirect effects through metabolites of microbiomes, (2) direct effects by impacting genomic stability of DNA damage and signaling related to host cell proliferation and death, and (3) direct effects by guiding immune system function^20^. In breast cancer, the metabolism of estrogen by the gut microbiota, which is termed the “estrobolome“, has set a foundation for the role of the gut microbiota. However, the concept of the estrobolome mainly exists in postmenopausal breast cancer patients. Little is known about the role of gut microbiota in premenopausal breast cancer patients.

The clinical and pathological features of premenopausal breast cancer differ from those of postmenopausal breast cancer. Premenopausal breast cancer is more aggressive and has a poorer prognosis than postmenopausal breast cancer^5^. In Asian countries, the high proportion of premenopausal breast cancer is a critical clinical issue that differs from the situation in Western countries. In addition, the luminal molecular subtype of premenopausal breast cancer in Asian countries, such as Taiwan, is more prevalent than in Western countries, where the basal-like subtype with a poor prognosis is dominant^21^. In our study of 200 breast cancer patients, the proportion of patients with premenopausal breast cancer was 50%, with greater tumor size and nearly the same distribution of grade and stage as that in patients with postmenopausal breast cancer. The molecular subtype of premenopausal breast cancer was true, with a high proportion of the luminal subtype, which accounted for more than 80% of cases.

In this study, we found that the α-diversity of the Shannon index was significantly reduced in premenopausal breast cancer patients compared with that in premenopausal controls. The phenomenon of lower α-diversity was unique to premenopausal breast cancer patients but not observed in postmenopausal breast cancer patients, whereas the α-diversity in postmenopausal controls was reduced compared with that in premenopausal controls. To date, only one study has investigated the gut microbiota in breast cancer patients and control individuals with different menopausal statuses. The researchers found that α-diversity was higher in breast cancer patients than in control individuals^22^. However, in our study, we provided a comprehensive analysis in which a lower α-diversity was specifically observed in premenopausal breast cancer.

The α-diversity is a general indicator used to evaluate the condition of the gut environment, including richness and evenness. Decreased diversity is generally correlated with aging, an unhealthy gut environment and disease status. This phenomenon of decreased diversity, termed loss of microbiota diversity (LOMD), is frequently associated with dysbiosis, which is the imbalance and abnormalities of the microbiota that result in negative effects on the host. LOMD is a general feature associated with many diseases and can even predict treatment response^23^. Herein, we also found a lower diversity in postmenopausal controls than in premenopausal controls due to the aging process. Several human studies have focused on the gut microbiota in terms of gender or menopausal status of healthy controls but not in breast cancer patients and demonstrated that α-diversity was lower in postmenopausal women^24,25^. However, there was no significant difference in α-diversity between premenopausal breast cancer patients and postmenopausal breast cancer patients, indicating that the LOMD was specific in premenopausal breast cancer without an age effect. There was a significant difference in the β-diversity of total microbial composition among the different menopausal statuses of breast cancer patients and controls. A study by Jia Zhu et al. also showed that the relative abundance of the gut microbiota did not differ significantly between premenopausal breast cancer patients and control individuals. Thus, the study by Jia Zhu et al. mainly focused on postmenopausal breast cancer^22^. However, our study provides the first evidence that gut microbiota profiles differ significantly according to menopausal status of breast cancer patients and control individuals.

To assess the differences in the total microbial composition, we conducted multiple analyses, including relative abundance of OTUs, LEfSe, Venn diagram, and heatmap assessments. The OTUs from the above analysis were reconfirmed by the RDP classifier to determine the critical microbial markers at the genus/species levels. Age is one of the crucial factors that need to be considered with regard to gut microbiota profiles, and gut microbiota patterns will change with age (i.e., from infants to elderly patients)^9,26^. Age differs between premenopausal and postmenopausal women, which is accompanied by different patterns of gut microbiota^24,25^. Thus, we carried out a comprehensive analysis to identify microbial markers that met the following criteria: (1) critical microbial markers that are not affected by age in premenopausal breast cancer patients, (2) microbial markers that fluctuate with age but are more obviously altered in breast cancer patients, and (3) universal microbial markers in breast cancer patients.

Five microbial markers were identified in premenopausal breast cancer patients. *Bifidobacterium* spp. are typical probiotics related to the maintenance of human health and exhibit reduced abundance with aging. Thus, the proportion of *Bifidobacterium* spp. was lower in postmenopausal controls. However, *Bifidobacterium* spp. were specifically reduced in premenopausal breast cancer patients, which was not observed in postmenopausal breast cancer patients. *Bifidobacterium* spp. are well known for their beneficial effects and disease prevention^27^. In cancer, *Bifidobacterium* spp. also play a critical role in tumor suppression through the immunomodulation and inhibition of DNA damage. The antitumor/proliferative effect of *Bifidobacterium* spp. against breast cancer was also observed in vitro and in vivo^28,29^, indicating the loss of tumor-suppressor-like *Bifidobacterium* spp. in premenopausal breast cancer patients. *Anaerostipes* has been reported to increase in endometrial cancer, hepatocellular carcinoma, and thyroid cancer^30–32^. In addition*, Bacteroides fragilis* is an oncobiome that contributes to colorectal cancer formation by inducing inflammation and DNA damage^33^. In breast cancer, a critical study demonstrated that *Bacteroides fragilis* exists in breast tissues and colonizes the gut to promote breast tumorigenesis and metastatic progression through the axes of the Notch and β-catenin pathways^34^. In postmenopausal breast cancer patients, *Akkermansia muciniphila* functions as a probiotic in individuals with obesity and diabetes. Several human studies have shown that the abundance of *Akkermansia muciniphila* decreases in individuals with metabolic diseases, such as obesity, diabetes, and hypertension^35,36^. The increasing abundance of *Akkermansia muciniphila* with aging in postmenopausal controls needs further investigation; however, *Akkermansia muciniphila* was found to be more abundant in elderly people than in young adults^37^. Furthermore, a slightly lower abundance of *A. muciniphila* was observed in Chinese centenarians than in Chinese elderly subjects^38^. In breast cancer patients, a high abundance of *Akkermansia muciniphila* was correlated with lower fat mass and higher diversity^39^. *Phascolarctobacterium* can produce short-chain fatty acids (SCFAs) and is decreased in individuals with many cancer types^31,40^. Regarding specific microbes in postmenopausal breast cancer patients, *Proteobacteria* is a phylum enriched with pathogenic bacteria and is a marker of dysbiosis/disease^41^. *Klebsiella pneumoniae* is also a pathogenic bacterium that elevates risk and promotes colorectal cancer development through the bacterial toxin colibactin^42^. Of the universal microbial markers, *Faecalibacterium prausnitzii* is a critical microbial marker for the generation of SCFAs and shows an antitumor effect in breast cancer^43^. In addition, *Faecalibacterium prausnitzii* is an index of dysbiosis, which indicates lower diversity and loss of tumor-suppressor-like microbes in premenopausal breast cancer patients^44^. *Ruminococcus gnavus* is a Crohn’s disease-related microbe that is reduced in gastrointestinal cancer^45,46^. *Rothia mucilaginosa* mainly exists in the oral cavity and is reduced in several cancer types^47,48^. The upregulation of *Sutterella* and *Haemophilus parainfluenzae*, which are pathogenic bacteria, is associated with autism, ulcerative colitis, and oropharyngeal cancer^47,49,50^.

To further clarify which microbial marker is critical in breast cancer according to age fluctuation, especially in premenopausal young breast cancer patients. Two populations, control and breast cancer populations, were divided according to the same age parameters. Correlation analysis provided critical evidence of two species of *Bacteroides fragilis* in young women with premenopausal breast cancer, which provides the first clinical evidence and is supported by the findings of Parida et al.^34^. Another species of *Klebsiella pneumoniae* in postmenopausal women with older breast cancer requires further investigation. In addition to microbial markers of the different menopausal statuses of breast cancer patients, we provided a novel noninvasive approach with a combination of multiple microbial markers for early detection of the different menopausal statuses of breast cancer patients. The concept of screening microbiomes of noninvasive specimens could be a future strategy for the diagnosis or prevention of the disease and has been indicated for many cancer types^22,31,51^.

In functional pathway analysis, microbes of premenopausal breast cancer patients were found to be involved in steroid-related aromatic and androstenedione degradation. Aromatic amines are byproducts of the manufacturing of rubber, industrial chemicals, dyes, etc. Females exposed to aromatic amines, especially during puberty and childbirth, have an increased risk of DNA damage and the development of breast cancer^52,53^. In the pathway of androstenedione degradation, which is converted to estrogen via the aromatase enzyme in the ovaries of premenopausal women. In addition to steroid-related pathways, the oncogenic-related pathway may be partially related to the activation of the Notch and β-catenin pathways of *Bacteroides fragilis*^34^. The microbiome of postmenopausal breast cancer patients is involved in chemical carcinogenesis and aldosterone-related pathways. In postmenopausal women, aldosterone levels will increase due to lower estrogen levels and may be involved in breast cancer^54^.

In fact, the involvement of the gut microbiota in breast cancer was proposed in 1981, when constipation was found to increase the risk of breast cancer^55^. The gut microbiota is dynamic and an adjustable target in which diet, probiotics, and prebiotics could reduce the risk of breast cancer^56^. The therapeutic goal is termed “rebiosis”, which means restoring a healthy and highly diverse microbial environment^23^. A meta-analysis of a prospective study showed that total fiber (especially soluble fiber) consumption was associated with an 8% lower risk of breast cancer^57^. Moreover, in a study of an Asian population, regular consumption of *Lactobacillus casei Shirota* (BLS), soy isoflavone, and vitamin D reduced breast cancer risk in premenopausal women, whereas alcohol consumption was significantly associated with a higher risk of breast cancer, specifically in premenopausal women^58–60^.

In conclusion, we comprehensively analyzed the microbial profiles, diagnostic values, and functional pathways in breast cancer patients with different menopausal statuses. In addition, we provided the first evidence that the gut microbiota in premenopausal breast cancer patients differs from that in postmenopausal breast cancer patients and revealed menopausal-specific microbial markers for diagnosis and investigation. Premenopausal breast cancer patients were characterized by a lower diversity of dysbiosis, a lower abundance of probiotics with tumor suppressors, DNA damage, and SCFA production, whereas postmenopausal breast cancer patients were characterized by dysbiosis of increasing pathogenic bacteria, providing a noninvasive approach for breast cancer detection and a novel strategy for preventing premenopausal breast cancer in the future.

## Supplementary information

Supplementary Information
