# Supplementary material for: Comprehensive profiles and diagnostic value of menopausal-specific gut microbiota in premenopausal breast cancer
Source: Exp Mol Med. 2021 Oct 27;53(10):1636–46. doi: 10.1038/s12276-021-00686-9 (PMC8569190; doi:10.1038/s12276-021-00686-9)

**Supplementary Information**

**Supplementary Figure 1** Microbial markers in patients with different receptor statuses of breast cancer. The 13 bacterial taxa did not show a significant trend among the different receptor statuses (luminal, HER2, basal), with only a small proportion of bacterial taxa showing statistical significance.

**Supplementary Figure 2** Microbial markers in the different stages of breast cancer. The 13 bacterial taxa did not show a significant trend among the different stages (stage 1, 2) with only a small proportion of bacterial taxa displaying statistical significance.

# Supplementary Fig. 1

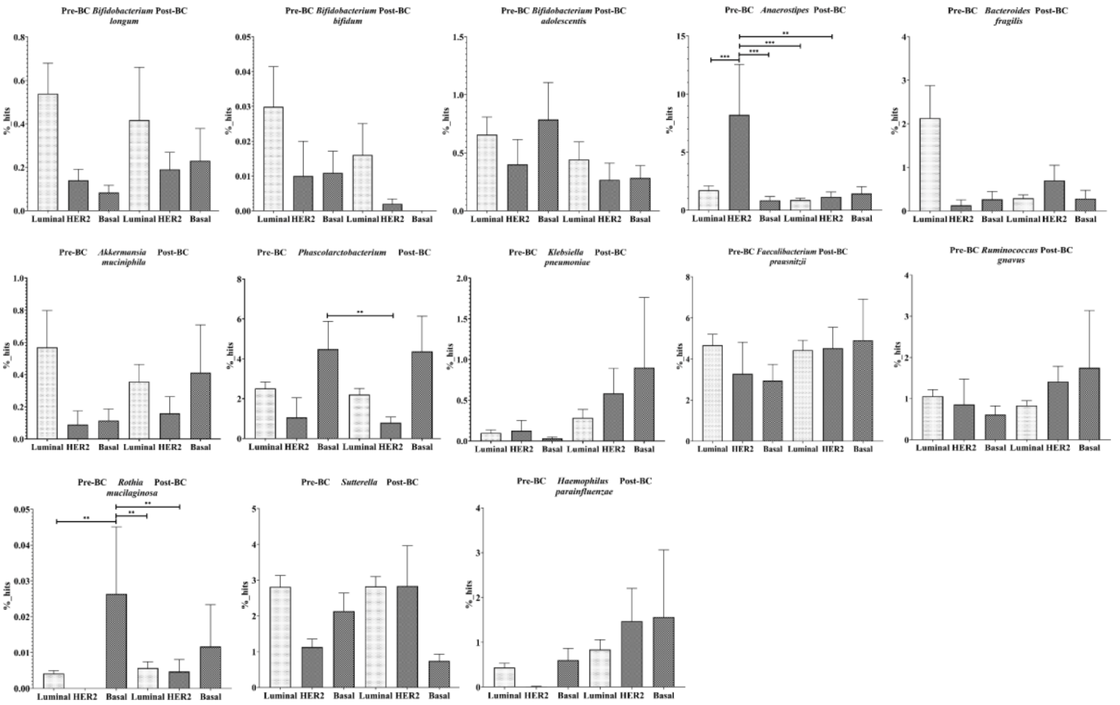

# Supplementary Fig. 2

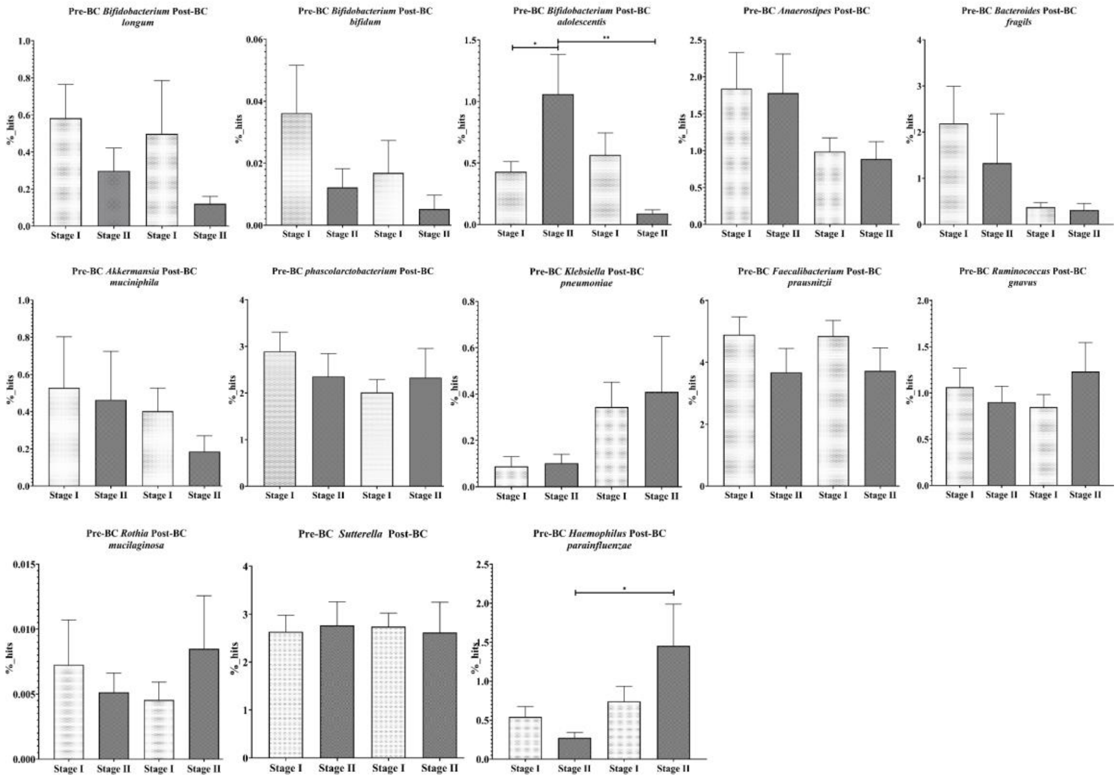

Supplement: Supplementary file 1 — Supplementary Information [file 12276_2021_686_MOESM1_ESM.pdf]
